# An ultra-rare case of immunoskeletal dysplasia with neurodevelopmental abnormalities in an Indian patient with homozygous c.953C > T variant in *EXTL3* gene: a case report

**DOI:** 10.1186/s12887-022-03143-2

**Published:** 2022-02-03

**Authors:** Shruti Bajaj, Purnima Satoskar, Aadhira Nair, Frenny Sheth, Jayesh Sheth, Harsh Sheth

**Affiliations:** 1 The Purple Gene Clinic, Simplex Khushaangan, SV Road, Malad West, Mumbai, 400064 Maharashtra India; 2 Department of Obstetrics and Gynaecology, Nowrosjee Wadia Maternity Hospital and Seth G. S. Medical College, Acharya Donde Marg, Parel, Mumbai, 400012 India; 3 FRIGE’s Institute of Human Genetics, FRIGE House, Jodhpur Gam Road, Satellite, 380015 Ahmedabad, India

**Keywords:** Bone dysplasia, Developmental delay, Heparan sulphate, Immunoskeletal dysplasia with neurodevelopmental abnormalities, EXTL3

## Abstract

**Background:**

Immunoskeletal dysplasia with neurodevelopmental abnormalities (ISDNA) is an ultra-rare genetic condition that belongs to the group of spondyloepimetaphyseal dysplasias. It is caused due to presence of biallelic variants in the *EXTL3* gene. The encoded exostosin like glycosyltransferase 3 (EXTL3) protein plays a key role in heparan sulfate synthesis. The skeletal and nervous systems are prominently affected in ISDNA with variability in immunological manifestations. Here, we report the 15^th^ case of ISDNA (third patient of an Indian ancestry) in the world, along with a review of literature.

**Case presentation:**

A 15-month-old female child with clinical indications of global developmental delay, short stature, coarse facial features, and hypotonia was referred to our clinic. Spondyloepimetaphyseal dysplasias associated with extra-skeletal manifestations was suspected based on clinic-radiological correlation. Whole exome sequencing analysis revealed the presence of a homozygous known pathogenic variant c.953C > T (p. Pro318Leu) in exon 3 of the *EXTL3* gene, thereby confirming diagnosis of ISDNA.

**Conclusion:**

We present an ultra-rare case of ISDNA- third patient of Indian ancestry and only the 15^th^ reported case in the literature. On review of all cases in the literature, we find that the affected individuals show abnormalities primarily in three systems namely- skeletal, nervous and immune system. Notably, patients harbouring the same variant in *EXTL3* gene show phenotypic variability especially with respect to presence or absence of immunological manifestations, suggesting a role of unknown modifiers. Hence, it is currently not possible to correlate the variant position in the *EXTL3* gene with disease severity.

## Background

Skeletal dysplasias comprise a large group of monogenic disorders primarily involving the skeletal system and accounting for at least 436 diseases [1]. A sub-group of diseases under this category involve immune dysfunction along with skeletal dysplasia, for example, cartilage-hair hypoplasia (Online Mendelian Inheritance of Man; OMIM#250,250), Schimke immuno-osseous dysplasia (OMIM#242,900), spondyloenchondrodysplasia with immune dysregulation (OMIM#607,944) and immunoskeletal dysplasia with neurodevelopmental abnormalities (ISDNA) (OMIM#617,425) [2–4]. Due to the progressive immune dysfunction in these conditions, there is a high mortality rate observed owing to increased susceptibility to infections and pulmonary disease [5].

ISDNA is an ultra-rare, autosomal recessive condition caused due to biallelic mutations in the *EXTL3* gene. It is consistently associated with skeletal features viz disproportionate skeletal dysplasia, odontoid hypoplasia, platyspondyly, epiphyseal and metaphyseal changes and more variably associated with immunodeficiencies and neurological features [6]. *EXTL3* gene is a member of the exostosin family that encode for exostosin like glycosyltransferase 3 protein, which is critical in the glycosylation process through which glycans are attached to both proteins and lipids in the endoplasmic reticulum or Golgi complex. The *EXTL3* gene (OMIM#605,744) located on chromosome 8p21.2 consists of 7 exons and encodes a protein of length 919 amino acids. There are two glycosyltransferase domains GT47 and GT64, of which the latter has both GlcNac transferase I and II activities [7].

To date, ISDNA has been described through the elaboration of 14 cases, incidentally reported in the same year (in 2017), by three separate groups [4, 6, 8]. Here, we describe the 15^th^ case of ISDNA (third patient of an Indian descent) harbouring a previously identified pathogenic variant in the *EXTL3* gene along with a review of the literature. This case adds to the existing repertoire of ISDNA cases and emphasizes the need for sequencing-based studies to provide accurate molecular diagnosis in cases of suspected spondyloepimetaphyseal dysplasias (SEMD).

## Case presentation

A third-degree consanguineous couple of Indian descent was presented for prenatal genetic counselling at eight weeks of gestation. Their older 15-month-old female child, had a suspected undiagnosed genetic disorder.

The index case was delivered at term after an uneventful antenatal period. The mother reported her third trimester scans suggestive of the foetus to have short long bones. Detailed medical or anthropometric records at birth were not available. The parents noted the child to have short stature, developmental delay and a dysmorphic face at three months of age. On assessment at 15 months, the child had global developmental delay. She had not attained head control, although she had achieved an approach to toys and mouthing (11 months), transfer of objects was missing. She could only coo (10 months), with no babbling achieved. She could recognise her parents (6 months), depicted social smile (7 months) and separation anxiety (11 months). Anthropometric charting on World Health Organisation growth charts was as follows: weight 6.7 kg (< -3 Z score), length 68 cm (< -3 Z score) and head circumference 43.5 cm (-1 to -2 Z score). The child had disproportionately short limbs (upper segment: lower segment 1.9:1). The facial features were coarse: full cheeks, frontal bossing, periorbital puffiness, depressed nasal bridge, prominent nose, long philtrum, anteverted nares (anteriorly-facing nostrils viewed with the head in the Frankfurt horizontal and the eyes of the observer level with the eyes of the subject) and a wide mouth. Other examination findings included: bilateral wrist widening, trident hands, gibbus in the dorsal spine region, signs of pigment dilution (light golden hair, grey irides, skin much fairer than the parents) and sparseness of scalp hair. The child had central hypotonia with normal deep tendon and plantar reflexes. The elbow extension was limited, while the wrist joints demonstrated hyperlaxity. Remaining examination was unremarkable.

**Fig. 1:**
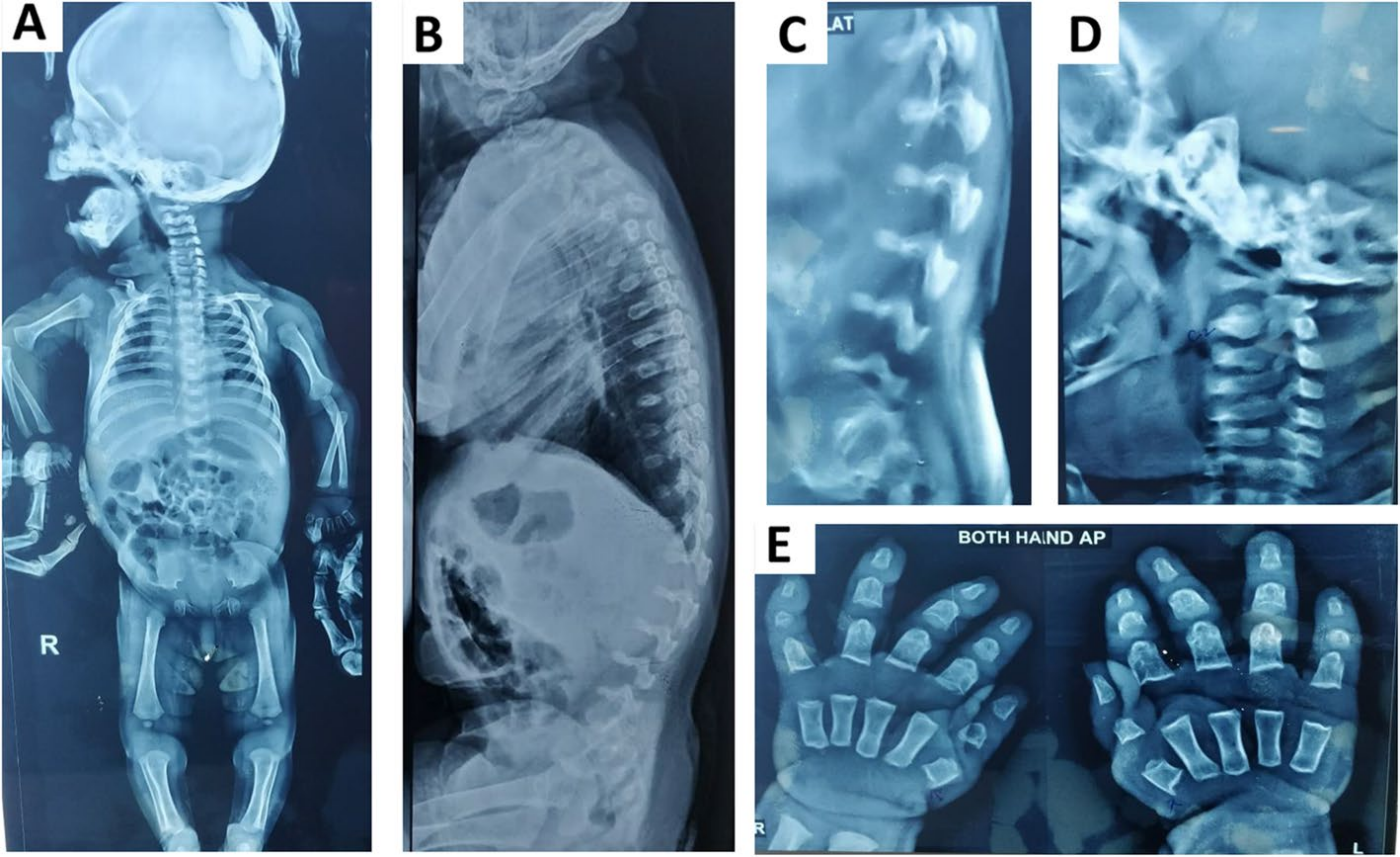
Radiographic images at 4 months (**a-e**). Full body x-ray (anteroposterior view)- short long bones, stout dumbbell-like long bones, along with convex metaphyseal margins and constriction at the mid-humerus, squared iliac bones with trident pelvis (‘snail-like’ pelvis) (**a**), full spine X-ray (lateral view)- platyspondyly of the cervical and thoracolumbar vertebrae (**b**), magnified x-ray view of the lower lumbar lateral view: severe platyspondyly with increased intervertebral spaces (**c**), lateral x-ray view of the cervical spine: platyspondyly, odontoid hypoplasia (**d**), X-ray of bilateral wrists (anteroposterior view)- brachydactyly and delayed bone age (**e**)

The radiographs done at 4-months revealed platyspondyly with increased intervertebral spaces, odontoid hypoplasia, squared iliac bones with trident pelvis (‘snail-like’ pelvis), coxa valga, brachydactyly and delayed bone age. Short, stout dumbbell-like long bones were appreciated, along with convex metaphyseal margins and constriction at the mid-humerus (Fig. 1a-e). Review of records revealed normal complete blood count, serum calcium and phosphorus (4 months) and a small fenestrated ventricular septal defect on 2-D echocardiogram (4 months). Cervical CT scan at 4 months showed non-visualisation of the ossification centres of the odontoid process and the anterior arch of the atlas, with suspicious ligamentous thickening and hypertrophy. The posterior arch of the atlas was represented by soft unossified tissue, with possibility of anterior displacement; raising the possibility of significant compromise of the spinal canal. Detailed ophthalmological assessment (4 months, 14 months), audiometric evaluation (4 months), quantitative and qualitative urinary glycosaminoglycan profile (4 and 14 months), and pelvic and abdomen sonogram, were reported normal. On detailed enquiry, there was no relevant family history in this case. Our clinical suspicion included spondyloepimetaphyseal dysplasias (SEMD), associated with extra-skeletal manifestations. Our differential diagnosis included odontochondrodysplasia (OMIM#184,260) and opsismodysplasia (OMIM#258,480). The couple was counselled about the need for a precise molecular diagnosis in a ‘proband-first’ approach, in order to guide them with robust prenatal counseling and testing in the current pregnancy.

Genomic DNA was extracted from peripheral blood of the proband using desalting protocol [9] and subjected to clinical exome sequencing. Target enrichment was performed using a custom capture kit targeting exons and exon–intron boundaries of approximately 4000 genes; the libraries were sequenced to mean > 80-100X coverage on the Illumina HiSeq sequencing platform (Illumina, USA). The obtained reads were aligned to the human reference genome assembly (GRCh37/hg19) using BWA [10] and germline variants were called using GATK v3.6 [11, 12]. Variant annotation was performed using VEP [13] against the Ensemble release 87 human gene model [14]. Variant filtration and prioritisation analysis revealed a homozygous variant c.953C > T (p.Pro318Leu) in exon 3 of the *EXTL3* gene (NM_001440.4). The variant was classified as likely pathogenic as per the ACMG-AMP guidelines and ClinGen framework [15–17] with following criteria – PM2 (moderate), PP2 (supporting), PP3 (supporting) and PP5 (supporting). This confirmed the genetic diagnosis of ISDNA in the proband. It was advised to perform Sanger sequencing to confirm the variant in the proband as well as to determine the carrier status in the parents, however the family was lost to follow-up and hence the analysis could not be carried out.

**Fig. 2:**
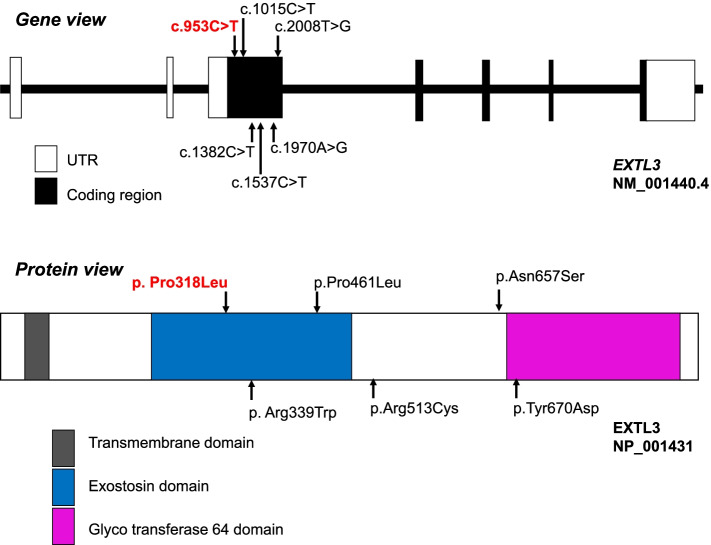
Schematic diagram of *EXTL3* and the corresponding protein with all the reported variants till date. *EXTL3* has a total of 7 exons and all variants are located in exon 3. The EXTL3 protein has a short transmembrane domain and two predicted Pfam domains: a conserved EXT domain and a glycosyl transferase family 64 domain. The variant identified in the proband is shown in red

We reviewed all the ISDNA cases reported in the literature and have summarised the clinical phenotype and genotype of the patients in Table 1. Out of the 10 families with affected ISDNA individuals, consanguinity was observed in four families. The common presenting symptoms included short limb dwarfism, platyspondyly, brachydactyly and epiphyseal abnormalities. Facial dysmorphism was observed in all patients. Interestingly, nine of the 14 patients of ISDNA (Table 1) had immunodeficiency and phenotype variability was documented amongst individuals harbouring the same variant in the *EXTL3* gene. All the variants reported in the *EXTL3* gene till date, are missense variants, located in exon 3 (Fig. 2). A total of five patients died before the age of one year, primarily due to recurrent infections.

**Table 1:** Overview of the phenotype and genotype of the 14 ISDNA cases in the literature along with the present case

Patient ID (Family)	Ethnicity	Consanguinity	Sex/Age^a^	Neurological phenotype	Immunological phenotype	Skeletal phenotype	Other	Outcome	Genotype
P1 (A)	Turkish	Yes	F/at birth	NA	T^−^ SCID	Short limb dwarfism, severe platyspondyly, metaphyseal changes, brachydactyly	Facial dysmorphism, liver cysts	Deceased at 7 weeks	c.1537C > T (p. Arg513Cys)
P2 (B)	Turkish	Yes	M/6 months	Severe ID	T^−^ SCID (Omenn like SCID)	Short limb dwarfism, epiphyseal abnormalities, brachydactyly	Facial dysmorphism	n.d	c.2008 T > G (p. Tyr670Asp)
P3 (B)	Turkish	Yes	F/at birth	Severe ID	T^−^ SCID (Omenn like SCID)	Short limb dwarfism, severe platyspondyly, epiphyseal abnormalities, brachydactyly	Facial dysmorphism	n.d	c.2008 T > G (p. Tyr670Asp)
P4 (C)	Columbian (South America)	Possibly	F/at birth	ID	None	Short limb dwarfism, severe platyspondyly, lumbar gibbus, kyphoscoliosis, epiphyseal abnormalities	Facial dysmorphism	n.d	c.1382C > T (p. Pro461Leu)
P5 (D)	Portuguese	Possibly	M/at birth	Borderline cognition	None	Short limb dwarfism, severe platyspondyly, lumbar gibbus, kyphoscoliosis, odontoid hypoplasia, cervical instability, epiphyseal abnormalities, brachydactyly	Facial dysmorphism	Deceased at 30 years	c.1382C > T (p. Pro461Leu)
P6 (D)	Portuguese	Possibly	F/at birth	None	None	Short limb dwarfism, severe platyspondyly, lumbar gibbus, kyphoscoliosis, odontoid hypoplasia, cervical instability, epiphyseal abnormalities, brachydactyly	Facial dysmorphism	n.d	c.1382C > T (p. Pro461Leu)
P7 (D)	Portuguese	Possibly	F/n.d	None	None	Short limb dwarfism, severe platyspondyly, lumbar gibbus, kyphoscoliosis, epiphyseal abnormalities	Facial dysmorphism	n.d	c.1382C > T (p. Pro461Leu)
P8 (E)	Indian	No	M/at birth	NA	Omenn like SCID	Short limb dwarfism, severe platyspondyly, odontoid hypoplasia, cervical instability, metaphyseal changes	Facial dysmorphism, liver cysts	Deceased at 6 months	c.1970A > G (p. Asn657Ser)
P9 (E)	Indian	No	M/at birth	NA	Omenn like SCID	Short limb dwarfism, severe platyspondyly, metaphyseal changes	Facial dysmorphism, liver cysts	Deceased at 10 months	c.1970A > G (p. Asn657Ser)
P10 (F)	Turkish	No	F/8.5 months	Motor delay, hypotonia, hyporeflexia	Recurrent pulmonary infections, dental caries, reduced IgM, IgG, T cells	Short limb dwarfism, elbow contractures, severe platyspondyly, kyphoscoliosis, pelvic dysplasia, broad thorax, broad ischia and pubes, constriction of proximal femora, brachydactyly, cord compression at craniovertebral junction	Facial dysmorphism, enlarged liver with few cystic lesions	n.d	c.953C > T (p. Pro318Leu)
P11 (G)	Turkish	Yes	F/8 months	Gross motor delay	Oral candidiasis	Short limb dwarfism, severe platyspondyly, kyphoscoliosis, pelvic dysplasia, constriction of proximal femora, brachydactyly, cord compression at craniovertebral junction	Facial dysmorphism, mitral valve prolapse, multiple liver cysts	n.d	c.953C > T (p. Pro318Leu)
P12 (H)	North African	n.d	M/at birth	Opisthotonos, hyperreflexia, seizures, DD, premature craniosynostosis	Omenn syndrome, hypogammaglobulinemia, increased IgE, sepsis, generalized exfoliative dermatitis,	Short limb dwarfism, severe platyspondyly, narrow sacro-ischiatic notches, trident-shaped, acetabula, short and plump limb bones, brachydactyly, cord compression	Severe narrowing of the laryngotracheal tract, liver cysts, death due to recurrent infections	Deceased at 11 months	c.1015C > T (p. Arg339Trp)
P13 (H)	North African	n.d	F/at birth	Clonic arm movements, nystagmus, developmental arrest, clover leaf skull	Sepsis, T^−^B^+^NK^+^SCID, hypogammaglobulinemia, increased IgE	Short limb dwarfism, severe platyspondyly, narrow sacro-ischiatic notches, trident-shaped, acetabula, short and plump limb bones, brachydactyly, cord compression	Severe narrowing of the laryngotracheal tract, liver cysts, anal atresia, death due to recurrent infections	Deceased at 7 months	c.1015C > T (p. Arg339Trp)
P14 (I)	Hispanic	No	F/at birth	Hypotonia, DD	Recurrent bilateral chalazion, recurrent blepharitis, T^−^B^+^NK^+^SCID Partial recovery documented in the course	Short limb dwarfism, severe platyspondyly, narrow sacro-ischiatic notches, trident-shaped, acetabula, short and plump limb bones, brachydactyly, cord compression	Facial dysmorphism, failure to thrive	n.d	c.1382C > T (p. Pro461Leu)
P15 (J)	Indian	Yes	F/15 months	DD, hypotonia	None	Short-limb dwarfism, trident hand, gibbus, elbow contractures, wrist laxity, snail-like pelvis, trident pelvis, severe platyspondyly, short stout long bones, convex metaphyseal ends, coxa valga, odontoid hypoplasia, delayed BA; cord compression at craniovertebral junction	Facial dysmorphism	^a^present case	c.953C > T (p. Pro318Leu)

^a^Age at presentation, *n.d.* no data

## Discussion and conclusions

*EXTL3* gene encodes a critical protein involved in the biosynthesis pathway of heparan sulphate (HS). Any impairment in the EXTL3 protein function due to an underlying mutation in the *EXTL3* gene disrupts the HS synthesis and causes a type of skeletal dysplasia- ISDNA. It is an ultra-rare genetic disorder with only 14 cases having been described in the literature to date. The key clinical indications include spinal cord compression, skeletal abnormalities like platyspondyly, brachydactyly and severe kyphosis. Deregulation of the immune system is another feature observed in this condition but is variable. The affected individuals experience severe morbidity due to the multisystem involvement.

The different effects of the mutant EXTL3 protein have been demonstrated through experimental studies in model organisms. In *extl3* gene mutant zebrafish, defective cartilage development and pectoral fin formation was observed [18–20]. Furthermore, these zebrafish had impaired thymic development and HS was observed to be critical for thymopoiesis [8]. These observations could explain the skeletal and immunological manifestations seen in patients affected with *EXTL3* gene mutations in humans.

The variant p.P318L identified in the present case has previously been identified in two Turkish individuals affected with ISDNA [4]. The amino acid residue at this position is highly conserved among diverse species. Additionally, in vitro functional studies have shown that there is loss of enzyme activity due to the presence of this mutation [4]. Both of these patients described earlier by Guo et al.had overlapping clinical features including spinal cord compression, craniofacial features and skeletal abnormalities like platyspondyly and brachydactyly. Also, severe immune deficiency was seen in one patient and not in the other. The skeletal features seen in our proband are consistent with those reported by Guo et al., however, there is a lack of immunodeficiency in our case. Following the genetic test, we couldn’t carry out reverse phenotyping which included assessment of the T-lymphocyte levels, a major diagnostic feature of ISDNA [6], due to the family's financial constraints. Interestingly, the present case did not show any liver abnormalities at the four-month sonogram although, we could not repeat the same at 15 months. This is in contrast to the previously described cases where enlarged liver and multiple liver cysts were noted [4]. It is possible that these manifestations may develop eventually, although that would require longitudinal follow-up which is beyond the scope of the present study. On comparing the clinical features of the proband with that of the reported ISDNA cases, we found facial dysmorphism and skeletal manifestations like short limb dwarfism, platyspondyly, brachydactyly, gibbus were consistent. However, the remaining gamut of immunodeficiency, neurological features, liver cysts, and spinal cord compression is relatively more variable, suggesting role of genetic and/or environmental modifiers [4, 6].

SEMD includes a group of clinically heterogeneous, lethal and non-lethal disorders under the larger classification of osteochondrodysplasias [21]. Amongst the SEMD group, two disorders can closely mimic ISDNA- odontochondrodysplasia and opsismodysplasia. They share certain common features viz neonatal short-limb dwarfism, platyspondyly, brachydactyly and non-lethality. Evolving mesomelia, dentinogenesis imperfecta, joint laxity, narrow thorax, genu varum and irregular metaphyseal flaring are associated with the former [22], whereas the latter is associated with facial dysmorphism (prominent brow, depressed nasal bridge, a small anteverted nose, and a relatively long philtrum), relative macrocephaly, severe hypotonia, delayed bone age and irregular metaphyseal ends [23]. Despite deep phenotyping, a significant phenotypic and radiographic overlap in this group of disorders makes molecular diagnosis clinically relevant.

Other than the genetic counselling, establishing a precise diagnosis had relevance for the medical care and surveillance of the proband; potentially impacting the quality and longevity of the life for the index case. The family was counselled about the likely need for spinal decompression surgery based on old radiological reports and was referred to neurosurgery services for assessment. However, the proband and the parents were lost to follow-up. Knowing the natural history of ISDNA, we would have liked to offer routine immune-surveillance and if indicated, intravenous immunoglobulins [4, 6, 8]. The child could have benefited from keen multidisciplinary care involving neurosurgeon, cardiologist, early intervention expert, neurologist, immunologist and geneticist [4, 6, 8].

Overall, our study reports the fifteenth ISDNA patient in the world, with a previously identified variant in the *EXTL3* gene. The proband shows a clinical presentation of skeletal dysplasia along with neuro-developmental delay which is similar to the previously described cases. Although all the affected individuals showed similar skeletal abnormalities, the variants in the context of genetic and/or environmental modifiers may have different effects on the function of the mutant EXTL3 protein, as there is variability noted in immunological phenotype. Therefore, it is difficult to correlate the disease severity only on the basis of the position of the variant. At present, there is no treatment available for this disorder except for supportive care and surgical interventions wherever possible. As more cases will be reported in future with simultaneous studies on the mutant EXTL3 protein, it would likely be possible to decipher the exact mechanism involved in the pathophysiology of this rare condition.

## Data Availability

Not applicable.

## Abbreviations

ISDNA: Immunoskeletal dysplasia with neurodevelopmental abnormalities
HS: Heparan sulphate
SEMD: Spondyloepimetaphyseal dysplasias
HSPG: Heparan sulfate proteoglycans
GlcNac: N-acetylglucosamine
